# Photoelectrochemical Water Splitting Properties of Ti-Ni-Si-O Nanostructures on Ti-Ni-Si Alloy

**DOI:** 10.3390/nano7110359

**Published:** 2017-10-31

**Authors:** Ting Li, Dongyan Ding, Zhenbiao Dong, Congqin Ning

**Affiliations:** 1 Institute of Electronic Materials and Technology, School of Materials Science and Engineering, Shanghai Jiao Tong University, Shanghai 200240, China; litingstar@sjtu.edu.cn (T.L.); dzb0312@126.com (Z.D.); 2 State Key Laboratory of High Performance Ceramics and Superfine Microstructure, Shanghai Institute of Ceramics, Chinese Academy of Sciences, Shanghai 200050, China; cqning@mail.sic.ac.cn

**Keywords:** anodization, TiO_2_ nanostructure, doping, photoelectrochemical water splitting

## Abstract

Ti-Ni-Si-O nanostructures were successfully prepared on Ti-1Ni-5Si alloy foils via electrochemical anodization in ethylene glycol/glycerol solutions containing a small amount of water. The Ti-Ni-Si-O nanostructures were characterized by field-emission scanning electron microscopy (FE-SEM), energy dispersive spectroscopy (EDS), X-ray diffraction (XRD), and diffuse reflectance absorption spectra. Furthermore, the photoelectrochemical water splitting properties of the Ti-Ni-Si-O nanostructure films were investigated. It was found that, after anodization, three different kinds of Ti-Ni-Si-O nanostructures formed in the α-Ti phase region, Ti_2_Ni phase region, and Ti_5_Si_3_ phase region of the alloy surface. Both the anatase and rutile phases of Ti-Ni-Si-O oxide appeared after annealing at 500 °C for 2 h. The photocurrent density obtained from the Ti-Ni-Si-O nanostructure photoanodes was 0.45 mA/cm^2^ at 0 V (vs. Ag/AgCl) in 1 M KOH solution. The above findings make it feasible to further explore excellent photoelectrochemical properties of the nanostructure-modified surface of Ti-Ni-Si ternary alloys.

## 1. Introduction

Titanium dioxide (TiO_2_) has been intensively investigated as a favorable, eco-friendly photocatalyst owing to its relatively low cost, nontoxicity, and stable chemical properties [1,2]. In 1972, TiO_2_ was used as a photochemical water splitting catalyst for the first time [3]. Recently, TiO_2_ was demonstrated to be a promising photocatalyst for photocatalytic water splitting and solar energy conversion with high efficiency and photochemical stability [4,5,6,7,8,9]. However, the wide energy band gap (3.2 eV for anatase and 3.0 eV for rutile) and the fast recombination of photogenerated electrons and holes are the main drawbacks of TiO_2_-based photoanodes [10]. Therefore, modification strategies including foreign element doping, surface decoration, and sensitization with dye have been adopted to overcome these drawbacks over the last 30 years [11,12,13,14,15,16]. One of the most studied methods is the doping of TiO_2_ materials with metal ions or nonmetallic elements such as Ni, Ta, Nb, Fe, Zn, C, N, and so on [17,18,19,20,21,22,23,24,25,26].

Ti-alloy-based oxide nanotubes were fabricated through a direct anodization of TiNi binary alloy [17,18]. To date, few studies have been conducted on the anodic fabrication of Ti-Ni-Si-O nanostructures on Ti-Ni-Si alloy substrates. Si has a much lower density than Ti (2.33 g/cm^3^ for Si vs. 4.54 g/cm^3^ for Ti) as well as vast natural abundance, and it is environmentally friendly. Zhang et al. [27] found that the presence of Si could impair the recombination of photogenerated electrons and holes effectively. Also, the photocurrent density of Si-doped TiO_2_ electrodes was 2–3 times higher than that of undoped TiO_2_ electrodes. In this work, Ti-Ni-Si-O nanostructures were successfully grown on Ti-Ni-Si ternary alloy substrates via electrochemical anodization in ethylene glycol/glycerol solutions containing a small amount of water. The microstructures and photoelectrochemical properties, especially the photochemical water splitting of Ti-Ni-Si-O nanostructures, were investigated.

## 2. Results and Discussion

**Figure 1:**
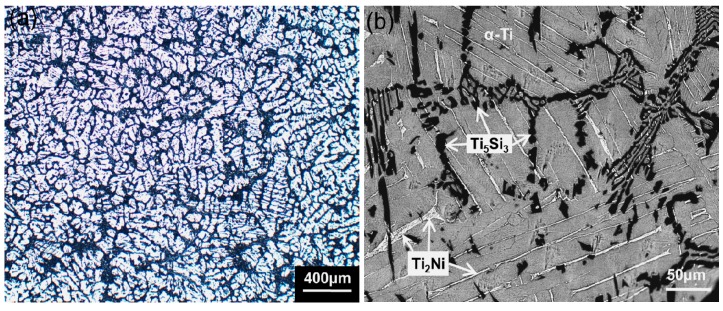
Typical microstructure of Ti-1Ni-5Si alloy: (**a**) Optical micrograph; (**b**) SEM image.

Figure 1 presents the typical microstructural features of as-cast Ti-1 wt % Ni-5 wt % Si alloy. Figure 1a shows the presence of multiphase, while Figure 1b shows a higher magnification image of different phases. EDS (energy dispersive spectroscopy) was used to test the compositions in the different phase regions. The EDS results are shown in Table 1. It was found that the gray region was α-Ti matrix, and the average composition of the black network-like region was 76.42 wt % Ti, 0.10 wt % Ni, and 23.48 wt % Si. Combined with the phase diagram calculated by Thermo-Calc software, it could be concluded that they were Ti_5_Si_3_ structures. In addition, the bright strip-like region was identified as the Ti_2_Ni phase [28]. It is noticeable that the quantity of the Ti_5_Si_3_ phase was much more than that of the Ti_2_Ni phase.

**Figure 2:**
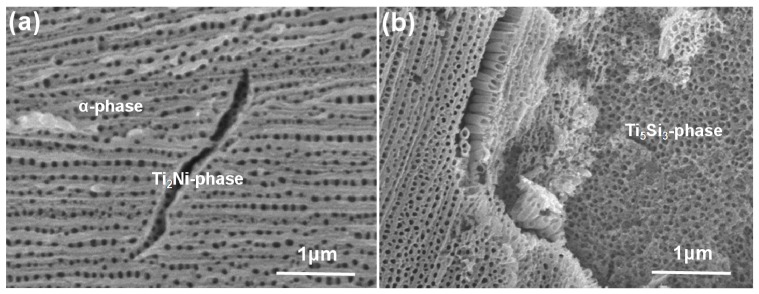
SEM images of scratched Ti-Ni-Si-O nanostructures showing: (**a**) nanotubes grown in the α-Ti phase region and Ti_2_Ni phase region; (**b**) nanopores grown in the Ti_5_Si_3_ phase region.

For the multi-phase Ti-1Ni-5Si alloy, the anodization process was not a uniform one due to the different anodization characteristics of different phases. Figure 2 shows SEM (scanning electron microscopy) images of different Ti-Ni-Si-O nanostructures grown in the α-Ti phase, Ti_2_Ni phase, and Ti_5_Si_3_ phase regions. Obviously, three kinds of nanostructures formed on the surface of the alloy films. One was a self-organized nanotube array formed in the α-Ti phase region. The second was a nanotube array under the corrosion pits in the Ti_2_Ni phase region. The third constituted irregular nanopores formed in the Ti_5_Si_3_ phase region. The Ti-Ni-Si-O nanotubes formed in the α-Ti phase region and the nanopores formed in the Ti_5_Si_3_ phase region had a pore diameter of about 64 nm. Table 2 shows the compositions tested by EDS for the α-Ti phase, Ti_2_Ni phase, and Ti_5_Si_3_ phase regions after anodization. It is noticeable that the Si element was still rich in the Ti_5_Si_3_ phase regions while the Ni element was relatively rich in the Ti_2_Ni phase regions.

The formation of TiO_2_ nanotubes by anodization can be roughly divided into two steps. In the first step, an initial barrier layer is formed on the electrolyte-metal interface. Then, an oxide barrier layer is randomly distributed by the chemical etching action of fluoride ions, resulting in the growth of nanotubes under the top oxide layer [29,30]. During the final step, the pore growth morphology gradually changes to a homogeneous and self-organized morphology. Thus, a competition between the formation and the dissolution of the oxides always takes place during the anodization process [31]. For the anodization of the Ti-Ni-Si alloy here, the Ti_2_Ni phase region and Ti_5_Si_3_ phase region should have a much quicker dissolution rate in the anodization electrolyte than the α-Ti phase region. For the Ti_2_Ni phase, the dissolution rate of the oxides was so fast that there was no time to form any nanostructures. Thus, only etching pits were left in this region. In the Ti_5_Si_3_ phase region, the dissolution rate was faster than the formation rate of the oxides; thus, it was difficult to form nanotube structures. Instead, nanopores formed in this region. With a slower dissolution rate in the α-Ti phase region, the formation of stable Ti-Ni-Si-O nanotubes became easier than that in the other phase regions. Our previous literature [32] reported the similar phase-dependent anodization of the two-phase Ti_6_Al_4_V alloy. Ti-Al-V-O nanotube arrays formed in the α-phase region and irregular Ti-Al-V-O nanopores formed in the V-riched β-phase region of the Ti_6_Al_4_V alloy. The solubility of vanadium oxide in the F^−^-containing electrolyte played an important role in the competition between the formation and dissolution of the oxides. It could be concluded that for the present anodization system, the phase-dependent anodization was hard to control for a uniform formation of nanotube arrays on top of the multiphase substrate.

**Figure 3:**
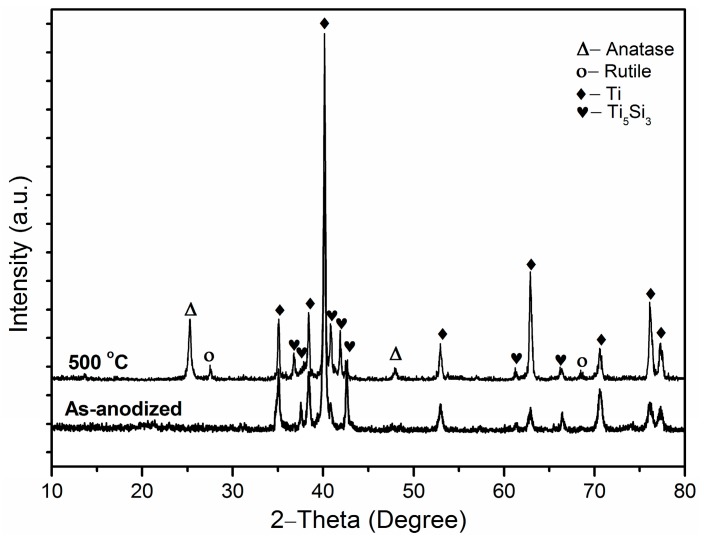
XRD patterns of the as-anodized and the annealed Ti-Ni-Si-O nanostructures.

The as-anodized Ti-Ni-Si-O nanostructures were found to be amorphous, and they could crystallize after the annealing process. XRD was adopted to determine the crystal structure and possible phases during annealing. Figure 3 presents the XRD patterns of the as-anodized and the annealed Ti-Ni-Si-O nanostructures. In the diffraction pattern of the annealed sample, two sharp diffraction peaks centered at 2θ angles of 40.2° and 62.9° were assigned to Ti metal (JCPDS card No. of 65-9622, Jade 5.0) from the substrate. The diffraction peaks at 25.3° and 75.0° could be assigned to the anatase phase (JCPDS card No. 21-1272, Jade 5.0) of TiO_2_. The peaks at 27.4° and 69.0° represented the rutile phase (JCPDS card No. 21-1276, Jade 5.0) of TiO_2_. The diffraction peaks at 36.8°, 40.8°, 41.9°, 42.6°, 61.2°, and 66.4° were indexed to the characteristic peaks of Ti_5_Si_3_ (JCPDS No. 65-3597, Jade 5.0) from the substrate. No diffraction peaks related to the Ti_2_Ni phase could be detected by XRD. In the diffraction pattern of the as-anodized sample, neither the anatase phase nor rutile phase could be observed. As shown in Figure 3, the amorphous structure of Ti-Ni-Si-O nanostructures had transformed into both anatase and rutile structures after annealing at 500 °C for 2 h. The anatase phase was found to be the major oxide phase. 

**Figure 4:**
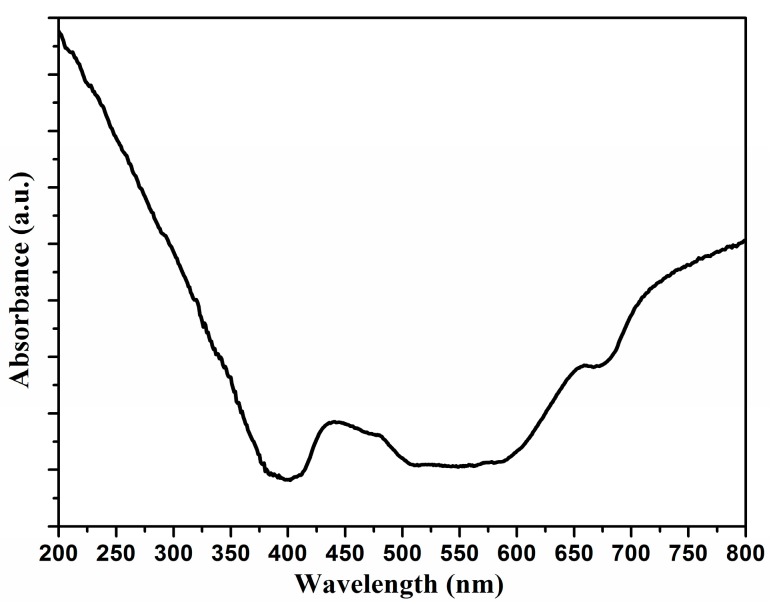
UV-Vis diffuse reflectance absorption spectra of the annealed Ti-Ni-Si-O photoanode.

Figure 4 shows the UV-Vis diffuse reflectance absorption spectra of the annealed Ti-Ni-Si-O photoanode. The band gap energy of the photoanode was estimated by using Tauc’s method. It was observed that the Ti-Ni-Si-O photoanode showed an absorption edge at 402 nm. The band gap value was 3.08 eV, which was between the anatase band gap (3.2 eV) and the rutile band gap values (3.0 eV). The presence of both the anatase structure and rutile structure was attributed to the obtained band gap value [11].

**Figure 5:**
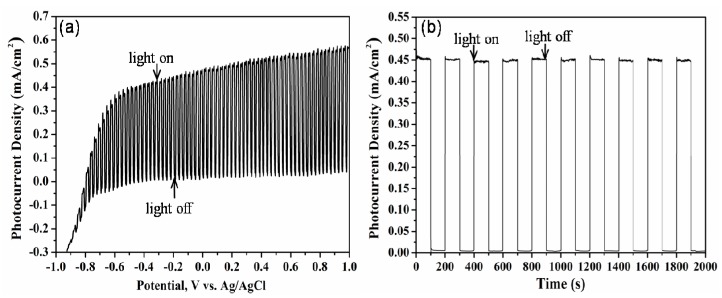
Photoelectrochemical water splitting behavior of Ti-Ni-Si-O nanostructures: (**a**) *I*-*V* curves in dark and under illumination; (**b**) transient photocurrent responses.

The photoelectrochemical water splitting behavior of Ti-Ni-Si-O nanostructures is shown in Figure 5. The linear sweep was collected for the Ti-Ni-Si-O photoanodes with a scan rate of 50 mV/s. The photocurrent density was 0.45 mA/cm^2^ at 0 V (vs. Ag/AgCl). The photocurrent under illumination was distinguishable from the dark current. Figure 4b presents photocurrent density vs. time scans for the Ti-Ni-Si-O photoanodes measured at 0 V (vs. Ag/AgCl). It could be seen that the photocurrent density was 0.45 mA/cm^2^, which was in accordance to the results of the linear sweep experiment. The samples demonstrated stable and instantaneous changes as well as reproducible responses in the photocurrent after many illumination on/off cycles.

**Figure 6:**
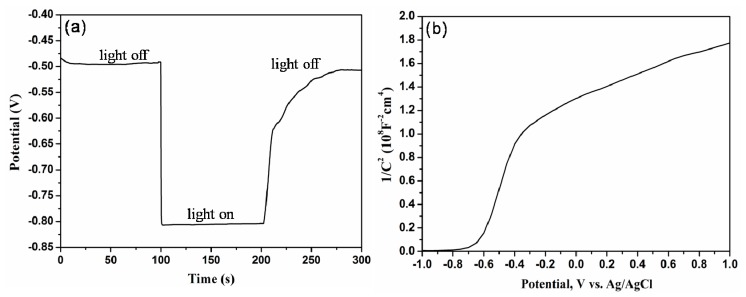
(**a**) Open-circuit potential of Ti-Ni-Si-O nanostructure photoanodes; (**b**) Mott-Schottky plots of Ti-Ni-Si-O nanostructure photoanodes with a frequency of 1000 Hz.

Electrochemical properties for Ti-Ni-Si-O nanostructure photoanodes annealed at 500 °C for 2 h were investigated, and the corresponding results are shown in Figure 6. Figure 6a shows the open-circuit potential (OCP) of Ti-Ni-Si-O photoanodes with time upon turning off the illumination. Without illumination, the OCP was about −0.50 V (vs. Ag/AgCl). As soon as the light was switched on, the OCP rapidly shifted negatively to a value of −0.80 V (vs. Ag/AgCl) due to the photogeneration of electron-hole pairs [33]. When turning off the illumination, the OCP gradually shifted positively to a steady state. These results indicated that the Ti-Ni-Si-O photoanodes had remarkable photoelectric conversion characteristics. The difference between the dark potential and light potential was about 0.3 V, which was the inherent characteristic of TiO_2_ [34].

The carrier concentration (*N_d_*) and flat-band potential (*V_FB_*) can be calculated from the Mott-Schottky equation [35,36]:

$$\frac{1}{C^{2}}=(\frac{2}{e_{0}{\varepsilon \varepsilon }_{0}N_{d}})[(V-V_{FB})-\frac{kT}{e_{0}}]$$

where *C* is the capacitance of the space-charge region, *e*_0_ is the electron charge (1.602 × 10^−19^ C), ε is the dielectric constant of TiO_2_ (ε = 41.4 for anatase TiO_2_ and 154.2 for rutile TiO_2_ [37]), ε_0_ is the permittivity of free space (8.854 × 10^−12^ F/m), *N_d_* is the donor density of N-type semiconductor (carriers/cm^3^), *V* is the applied potential bias at the electrode, k is the Boltzmann’s constant (1.38 × 10^−23^ J/K), and *T* is the absolute temperature. It can be seen that there is a linear relationship between 1/*C*^2^ and *V_FB_*. Furthermore, the flat-band potential *V_FB_* can be calculated from the extrapolation of the line to 1/*C*^2^ = 0. Moreover, the carrier concentration can be obtained from the slope of the Mott-Schottky equation. Figure 6b presents Mott-Schottky plots of Ti-Ni-Si-O photoanodes with a frequency of 1000 Hz. It was calculated that the flat-band potential was −0.625 V (vs. Ag/AgCl). The carrier concentration was in the range of 2.13 × 10^16^/cm^3^ to 8.57 × 10^16^/cm^3^ for Ti-Ni-Si-O photoanodes, which was comparable with those of pure TiO_2_ photoanodes [23]. Simelys et al. [38] reported that a higher carrier concentration could facilitate the charge separation at the semiconductor-electrolyte interface, and the carrier concentration reached up to 7.05 × 10^19^/cm^3^ for the TiO_2_ nanotubes with a thickness of about 1.5 μm. The samples here showed a positive slope in the Mott-Schottky plots, as expected for an N-type semiconductor.

## 3. Materials and Methods

Ti-Ni-Si-O oxide films on the alloy substrate were synthesized through a direct anodic oxidation process. Prior to the anodization, the Ti-1Ni-5Si alloy foils with a size of 20 mm × 10 mm × 1 mm were mechanically polished and ultrasonically degreased in acetone and ethanol, rinsed with deionized water, and finally dried in air. The anodization was carried out in a conventional two-electrode electrochemical cell with the alloy foil as a working electrode and the platinum foil as a counter electrode at room temperature. All of the samples were anodized at a pulse voltage of 40 V with a constant frequency of 4000 Hz and a duty cycle of 50% for 90 min in an electrolyte of 5 vol % ethylene glycol/glycerol (Shanghai Lingfeng Chemical Reagent Co., Ltd., Shanghai, China) containing 0.30 M (NH_4_)_2_SO_4_ and 0.4 M NH_4_F (Sinopharm Chemical Reagent Co., Ltd., Shanghai, China) as well as 3 vol % deionized water. After anodization, the samples were immediately rinsed with deionized water and subsequently dried in air. All of the samples were annealed at 500 °C for 2 h in air to transform amorphous oxide into crystalline phases.

The structure and morphology of the oxide film were characterized through field emission scanning electron microscopy (SEM, FEI SIRION 200, Hillsboro, OR, USA). The chemical compositions were analyzed by energy dispersive spectroscopy (EDS, INCA X-ACT, Oxford, UK). The crystalline phase was characterized with an X-ray diffractometer (Rigaku Ultima IV, Tokyo, Japan) with Cu K_α_ radiation (λ = 0.15406 nm) at 40 kV and 30 mA with a scan speed of 5°/min over a 2θ range from 10° to 80°. Diffuse reflectance absorption spectra were collected by a UV-visible spectrometer (Perkin Elmer Inc., Lambda 750S, Waltham, MA, USA) with BaSO_4_ as a reference. The photoelectrochemical measurement of different photoanodes was performed in 1 M KOH solution using a typical three-electrode system with oxide photoanode as a working electrode, Pt as a counter electrode, and Ag/AgCl as a reference electrode. A 150 W Xe lamp (Lanpu XQ350W, Shanghai, China) was used as a light source and the intensity of light illumination was controlled at 100 mW/cm^2^. The illuminated area of the working electrode was 1 cm^2^.

## 4. Conclusions

In summary, Ti-Ni-Si-O nanostructures were successfully fabricated through electrochemical anodization for photoelectrocatalytic water splitting. It was found that after anodization, three kinds of Ti-Ni-Si-O nanostructures grew in the α-Ti phase region, Ti_2_Ni phase region, and the Ti_5_Si_3_ phase region of the alloy surface. Both anatase and rutile structures of Ti-Ni-Si-O oxide appeared after annealing at 500 °C for 2 h. The photocurrent density obtained from the Ti-Ni-Si-O nanostructure photoanodes was 0.45 mA/cm^2^ at 0 V (vs. Ag/AgCl) in 1 M KOH solution. The above findings make it feasible to further explore the excellent photoelectrochemical properties of the nanostructure-modified surfaces of Ti-Ni-Si ternary alloys.

## Figures and Tables

**Table 1:** Compositions of the α-Ti phase, Ti_2_Ni phase, and Ti_5_Si_3_ phase of the alloy.

EDS Testing Areas	Elements (wt %)		
	Ti	Ni	Si
α-Ti phase	98.81	0.12	1.07
Ti_2_Ni phase	88.02	11.89	0.09
Ti_5_Si_3_ phase	76.42	0.10	23.48

**Table 2:** Compositions in the α-Ti phase, Ti_2_Ni phase, and Ti_5_Si_3_ phase regions after anodization.

EDS Testing Areas	Elements (wt %)			
	Ti	Ni	Si	O
α-Ti phase region	56.48	–	1.06	42.46
Ti_2_Ni phase region	66.77	1.94	0.82	30.47
Ti_5_Si_3_ phase region	60.35	–	9.74	29.91
